# Shared and unshared exposure measurement error in occupational cohort studies and their effects on statistical inference in proportional hazards models

**DOI:** 10.1371/journal.pone.0190792

**Published:** 2018-02-06

**Authors:** Sabine Hoffmann, Dominique Laurier, Estelle Rage, Chantal Guihenneuc, Sophie Ancelet

**Affiliations:** 1 Institut de Radioprotection et de Sûreté Nucléaire, Fontenay-aux-Roses, France; 2 Faculté de Pharmacie, Université Paris Descartes, Paris, France; North Carolina State University, UNITED STATES

## Abstract

Exposure measurement error represents one of the most important sources of uncertainty in epidemiology. When exposure uncertainty is not or only poorly accounted for, it can lead to biased risk estimates and a distortion of the shape of the exposure-response relationship. In occupational cohort studies, the time-dependent nature of exposure and changes in the method of exposure assessment may create complex error structures. When a method of group-level exposure assessment is used, individual worker practices and the imprecision of the instrument used to measure the average exposure for a group of workers may give rise to errors that are shared between workers, within workers or both. In contrast to unshared measurement error, the effects of shared errors remain largely unknown. Moreover, exposure uncertainty and magnitude of exposure are typically highest for the earliest years of exposure. We conduct a simulation study based on exposure data of the French cohort of uranium miners to compare the effects of shared and unshared exposure uncertainty on risk estimation and on the shape of the exposure-response curve in proportional hazards models. Our results indicate that uncertainty components shared within workers cause more bias in risk estimation and a more severe attenuation of the exposure-response relationship than unshared exposure uncertainty or exposure uncertainty shared between individuals. These findings underline the importance of careful characterisation and modeling of exposure uncertainty in observational studies.

## Introduction

Exposure measurement error is arguably one of the most important sources of uncertainty in epidemiological studies. It is widely acknowledged that when it is not or only poorly accounted for, measurement error can lead to biased risk estimates, a distortion of the shape of the exposure-response relationship and a loss in statistical power [1, 2]. Accounting for exposure measurement error can be daunting, however, because error characteristics tend to be complex in epidemiological studies.

In occupational cohort studies, for instance, one is usually interested in the association between the time until diagnosis or time until death by a certain disease and cumulative exposure to a certain chemical or physical agent. The analysis of this association may require the specification of a proportional hazards model where cumulative exposure is treated as a time-dependent variable. Owing to the time-dependent nature of cumulative exposure, the exposure history of a worker may be collected using different strategies according to the period of exposure. Changes in the methods of exposure assessment can create rather complex patterns of exposure uncertainty, where the type and magnitude of measurement error can vary over time. If no exposure data is available for the earliest years, one usually has to retrospectively reconstruct exposure values for this period. On the other hand, it is common to use a method of prospective, and possibly individual, exposure monitoring for the more recent exposure periods. In the periods of prospective exposure assessment, technical advances in measurement devices may imply more and more precise measures of exposure, which can translate into a decrease in measurement error over time. It has been suggested that the fact that exposure uncertainty and the magnitude of exposure are both highest for the earliest exposure periods may cause an attenuation of the exposure-response curve for high exposure values, a phenomenon frequently observed in occupational cohort studies [3–5].

As it is virtually impossible to reconstruct the exposure values for each individual worker in a retrospective fashion, one usually has to estimate the exposure levels for different job categories and the same exposure level is affected for all workers in a given job category. In this situation, individual exposure values of workers in a job-category are assumed to vary around the estimated exposure level and measurement error is therefore often described as unshared Berkson error [5–8], i.e., Berkson error that independently affects workers and different exposure values of the same worker. In this conception, the estimated exposure level is implicitly considered to be a precise estimate of the true average exposure in a job category, thereby neglecting the fact that many simplistic and potentially wrong assumptions typically have to be made in retrospective exposure reconstruction. Considering these uncertainties and simplifications, which often arise because working conditions may be very different from those in more recent years, the estimated exposure level can greatly differ from the true average exposure level in a job category. This discrepancy can be modelled as a classical measurement error component that is shared between workers. Indeed, it affects the exposure values of all workers in a given job category in the same way and therefore cannot be considered as independent for workers who belong to this job category. Several authors have described this error structure as a mixture of unshared Berkson and unshared classical measurement error [9, 10], but this view cannot account for the fact that the classical measurement error component affects all individuals in a group in the same way. At the same time, uncertainty components that are shared between individuals have received growing attention in the field of radiation epidemiology in recent years [11–15]. Quite contrary to [9, 10], the authors of [14, 15] account for the shared nature of error, but not for the fact that this shared error can be of Berkson or classical type [15, 16]. Meanwhile, comparatively little attention has been paid to the possibility of error components shared within workers in occupational cohort studies. When cumulative exposure is modelled as a time-dependent variable and a method of group-level exposure estimation is used, individual job conditions and worker practices may create a correlation between measurement errors in the exposure history of a worker [17, 18]. This correlation can be described by error shared within workers, i.e. an exposure uncertainty component shared for several years of the same worker. Through the summing of exposure values to obtain cumulative exposure, uncertainty components shared within workers may be magnified, as the same error term is repeated for every exposure value of a worker. Therefore these components may have more impact on statistical inference than unshared uncertainty components or components that are shared between workers. On the other hand, several authors have argued that errors that are shared between individuals might have fundamentally different consequences on statistical inference than unshared measurement errors [13–16]. To our knowledge there are no studies confirming this assertion for proportional hazards models, which possibly presents the most widely applied class of models in medical research.

Stayner et al. [4] and Steenland et al. [5] examined the effects of heteroscedastic measurement error on the shape of the exposure-response curve and only found a modest attenuation of the exposure-response curve at high exposure values. However, the authors treated cumulative exposure in an occupational cohort as time fixed variable known at baseline, thereby ignoring both its time-varying nature and the possibility of exposure uncertainty components shared within individuals. In this context, further analyses are necessary to reassess these results when a more realistic structure of measurement error is assumed with the possibility of shared and unshared uncertainty components for all exposure periods.

The aim of this study is to highlight and compare the effects of shared and unshared exposure measurement error on risk estimation and the shape of the exposure-response relationship with the aid of simulated data when statistical inference in proportional hazards models is not corrected for exposure measurement error. In a first step, we will assume measurement models with only one type of error for all periods of exposure to compare the impact of different types of exposure uncertainty on risk estimation in two alternative proportional hazards models. In particular, we will conduct a first simulation study, referred to as simulation study 1 in the following, to investigate the influence of multiplicative Berkson and classical measurement error that can be shared between workers, within workers, both between workers and within workers, or unshared. The aim of this first simulation study is thereby to assess whether shared error components have fundamentally different consequences on risk estimation than unshared error components. In a second simulation study, referred to as simulation study 2 in the following, we will assume more complex measurement models with varying types of multiplicative measurement error for different exposure periods that reflect the conditions in an occupational cohort more realistically to assess the effects of these error structures on the shape of the exposure-response relationship. The aim of this second simulation study is thereby to investigate the possibility of complex error structures to lead to an attenuation of the exposure-response relationship in an occupational cohort study.

Our motivating example concerns the potential impact of uncertainty in radon exposure when modelling lung cancer mortality in the French cohort of uranium miners.

## Measurement models

In the following, we present several measurement models to describe shared and unshared exposure uncertainty components in an occupational cohort, which we will use in our simulation study. These measurement models describe the association between the true *X*_*ij*_(*t*) and the observed *Z*_*ij*_(*t*) exposure of worker *i* at time *t*, where worker *i* belongs to group *j*. A group can be formed by all workers belonging to a specific job-category in a retrospective exposure reconstruction or in a prospective method of group-level estimation. For the sake of simplicity, we will assume that each worker can only belong to one group and that the group he belongs to does not vary over time.

A large part of the measurement error literature is based on additive error. As it has been repeatedly suggested, however, that multiplicative measurement models may be more realistic in many situations in occupational and environmental epidemiology in general [6] and to describe uncertainty in airborne exposure in particular [3, 7, 19], we will assume a multiplicative log-normal model for exposure measurement error in the following. In order to describe exposure uncertainty in an occupational cohort, we will first consider simple measurement models in which the type (i.e., Berkson, classical, shared, unshared) and magnitude of error remains constant over the years. In other words, even though there may be several exposure periods, we will consider the same measurement error variance for all exposure periods. We will refer to these models as homoscedastic measurement models. In a second step, we will focus on more complex measurement models that may describe exposure uncertainty more adequately in an occupational cohort. Contrary to the homoscedastic measurement models that assume pure shared or unshared measurement error for all exposure periods, we will consider in these complex models that the type of error and the measurement error variances can vary for the different exposure periods. Additionally, we will consider that measurement error occurring in a given exposure period can be characterised by a combination of shared and unshared exposure uncertainty components. We will refer to these models as heteroscedastic measurement models.

### Homoscedastic measurement models

#### Unshared measurement error

When modelling measurement error, one commonly distinguishes Berkson and classical measurement error. Unshared Berkson error is often presumed when a group-level method of exposure estimation is used. In this case, an observed or estimated exposure value is assigned to a group of workers and the true exposure of each worker is supposed to randomly deviate from this observed exposure value. The variability of true exposure is larger than the variability of observed exposure and measurement error is independent of observed exposure. Multiplicative Berkson error can be expressed by model

$$\begin{array}{c} M_{1}:X_{ij}\left(t\right)=Z_{j}\left(t\right)\cdot U_{ij}\left(t\right), \end{array}$$

where *Z*_*ij*_(*t*) = *Z*_*j*_(*t*) for all workers *i* of group *j* and *E*(*U*_*ij*_(*t*)|*Z*_*j*_(*t*)) = 1, which implies that *E*(*X*_*ij*_(*t*)|*Z*_*j*_(*t*)) = *Z*_*j*_(*t*).

When individual measurements are obtained through a measuring device, on the other hand, a classical measurement error model is assumed, where the observed exposure of worker *i* in group *j* *Z*_*ij*_(*t*) randomly deviates from his true exposure. In contrast to model $M_{1}$, the variability of observed exposure is larger than the variability of true exposure and measurement error is independent of true exposure. Multiplicative classical measurement error can be expressed by model

$$\begin{array}{c} M_{2}:Z_{ij}\left(t\right)=X_{ij}\left(t\right)\cdot U_{ij}\left(t\right), \end{array}$$

where *E*(*U*_*ij*_(*t*)|*X*_*ij*_(*t*)) = 1 implying that *E*(*Z*_*ij*_(*t*)|*X*_*ij*_(*t*)) = *X*_*ij*_(*t*). Contrary to measurement model $M_{1}$, we do not dispose of *E*(*X*_*ij*_(*t*)|*Z*_*ij*_(*t*)) in measurement model $M_{2}$. A common way to correct for measurement error in this situation is to use regression calibration, where *E*(*X*_*ij*_(*t*)|*Z*_*ij*_(*t*)) is modelled as a function of *Z*_*ij*_(*t*) and the parameters of this function are estimated on a validation sample.

We will assume in both models that log transformed measurement errors *log*(*U*_*ij*_(*t*)) are independent and normal random variables with mean $-\frac{\sigma^{2}}{2}$ and variance *σ*^2^, i.e., $\text{log}\left(U_{ij}\left(t\right)\right)∼N\left(-\frac{\sigma^{2}}{2},\sigma^{2}\right)$. Note that if log(*W*) follows a normal distribution with mean *μ* and standard deviation *σ*, *W* follows a log-normal distribution with $E\left(W\right)=\exp\left(\mu +\frac{\sigma^{2}}{2}\right)$ and variance *Var*(*W*) = (exp(*σ*^2^) − 1)exp(2*μ* + *σ*^2^). The chosen parametrisation thereby ensures that *E*(*U*_*ij*_(*t*)|*Z*_*j*_(*t*)) = 1 and *E*(*U*_*ij*_(*t*)|*X*_*ij*_(*t*)) = 1. In particular, *U*_*ij*_(*t*) and *U*_*i*′ *j*_(*t*′) are independent for *i* ≠ *i*′ and *t* ≠ *t*′ in both $M_{1}$ and $M_{2}$. Under this independence assumption, measurement error is considered as unshared.

#### Shared measurement error

To describe exposure uncertainty components that are shared between or within workers, one can adapt the unshared Berkson and classical measurement error model described previously by modifying the assumptions on the structure of measurement error *U*_*ij*_(*t*).

For instance, if we suppose that the measurement error term *U*_*ij*_(*t*) equals *U*_*j*_(*t*) for all workers *i* in group *j*, we can obtain a Berkson and a classical measurement error model in which the errors are shared for a group of workers. Indeed, in these models, the same error component is presumed at time *t* for all subjects *i* belonging to group *j* (hence the term “shared between workers”) and *U*_*j*_(*t*) and *U*_*j*′_(*t*′) are independent if *j* ≠ *j*′ or *t* ≠ *t*′. In an occupational cohort study, we will be faced with components of classical measurement error that are shared between workers in a situation where a measuring device is used to measure the mean exposure of a group of workers in a prospective fashion. A measurement error occurring on the measuring device will affect the exposure values of all workers in the same way and thereby lead to a classical measurement error component shared between workers. If, on the other hand, this group consists for instance of workers that work in three different locations, it is likely that the exposure levels in a given location are rather homogeneous while there can be an important heterogeneity between these three locations. The errors arising due to this heterogeneity may then adequately be described by Berkson error shared between all workers in a given working location. We will denote $M_{3}$ and $M_{4}$ the measurement models describing Berkson and classical measurement error shared between workers.

To describe Berkson or classical measurement error that is shared for several years of the same worker, we assume that the measurement error term *U*_*ij*_(*t*) neither depends on time *t* nor on group *j*: *U*_*ij*_(*t*) = *U*_*i*_ ∀*j*∀*t*. The same error component is supposed for all years of exposure of worker *i* and *U*_*i*_ and *U*_*i*′_ are independent if *i* ≠ *i*′ (hence the term “shared within workers”). A component of classical measurement error shared for several exposure years of the same worker may arise if each worker has a personal measuring device. If the measuring device of a worker is not perfectly calibrated, there may be a systematic error that affects all exposure values received by this worker in the same way. If a strategy of group exposure assessment is chosen, on the other hand, individual job conditions and worker practices can lead to a component of Berkson error that is shared for several years of exposure of the same worker. We will denote $M_{5}$ and $M_{6}$ the measurement models describing Berkson and classical measurement error shared within workers.

Finally, an error component may simultaneously affect all exposure values received by the workers in a certain group during an exposure period of several years in the same way. This error structure is likely to occur when recent exposure conditions are extrapolated in order to reconstruct exposure values in the past. These extrapolations are typically made for a group of workers and for a period of several years at the same time, as it is difficult to make more precise estimates in a retrospective exposure reconstruction. In this situation, the measurement error induced by estimating the mean exposure level for a group *j* affects both all workers in this group and all exposure values received by these workers. Therefore, the measurement error term *U*_*ij*_(*t*) neither depends on time *t* nor on subject *i*, but only on the group *j* a subject belongs to: *U*_*ij*_(*t*) = *U*_*j*_ ∀*i*∀*t*. In this case, *U*_*j*_ and *U*_*j*′_ are independent if *j* ≠ *j*′. We will denote $M_{7}$ and $M_{8}$ the measurement models describing Berkson and classical measurement error shared both within and between workers.

For a more detailed presentation of models $M_{3}$ to $M_{8}$, see S1 File.

### Heteroscedastic measurement models with three exposure periods

In the following, we will extend the measurement models presented so far by considering three exposure periods for which both the type and the magnitude of exposure uncertainty can vary and by allowing for combinations of shared and unshared measurement error in each exposure period. In this section, we denote $X_{ij}^{q}\left(t\right)$ and $Z_{ij}^{q}\left(t\right)$ the true and observed exposure for worker *i* belonging to group *j* at time *t* in exposure period *q*.

#### Unshared measurement error for different exposure periods

Motivated by the exposure conditions in the French cohort of uranium miners (see the motivating example described in the presentation of the simulation study for more details), we consider the following model to allow for possible changes in the method of exposure assessment:

$$\begin{array}{c} M_{9}:\{\begin{array}{c} X_{ij}^{1}\left(t\right)=Z_{j}^{1}\cdot U_{ij}^{1}\left(t\right)\\ X_{ij}^{2}\left(t\right)=Z_{j}^{2}\left(t\right)\cdot U_{ij}^{2}\left(t\right)\\ Z_{ij}^{3}\left(t\right)=X_{ij}^{3}\left(t\right)\cdot U_{ij}^{3}\left(t\right) \end{array} \end{array}$$

where $\text{log}(U_{ij}^{q}\left(t\right))$ are independent and normal random variables with mean $-\frac{\sigma_{q}^{2}}{2}$ and variance $\sigma_{q}^{2}$. We suppose three distinct exposure periods, *q* ∈ {1, 2, 3}, with a retrospective exposure reconstruction for the first period, a prospective method of group-level exposure estimation for the second period and prospective and individual exposure assessment for the third period. A similar reasoning will apply to many occupational cohort studies, as exposure values for the earliest years of exposure are often reconstructed retrospectively by extrapolating exposure conditions from more recent exposure periods, while methods of prospective exposure assessment are available for more recent years of exposure [3, 20, 21]. The models can also be easily extended to more than three exposure periods. By allowing $\sigma_{q}^{2}\neq \sigma_{q^{\prime }}^{2}$ for *q* ≠ *q*′, model $M_{9}$ can describe the varying type and magnitude of error in the three exposure periods by a heteroscedastic and unshared error structure. The error occurring in the first two exposure periods is described as Berkson error with $E(U_{ij}^{1}\left(t\right)|Z_{j}^{1})=1$ and $E(U_{ij}^{2}\left(t\right)|Z_{j}^{2}\left(t\right))=1$, following the assumption that a method of group-level exposure estimation will lead to unshared Berkson error. The observed exposure $Z_{j}^{1}$ for group *j* in the first period does not depend on time *t* as only one value is estimated for all exposure years in that group in a retrospective exposure reconstruction. On the other hand, observed exposure $Z_{j}^{2}\left(t\right)$ for group *j* in the second period depends on time *t*, as exposure values are estimated by a prospective group-level exposure assessment. The individual and prospective method of exposure assessment in the third exposure period, on the other hand, is supposed to produce independent classical measurement error, which implies that $E(U_{ij}^{3}\left(t\right)|X_{ij}^{3}\left(t\right))=1$.

#### Accounting for the imprecision of the measurement device in group-level exposure estimation

When describing exposure uncertainty in an occupational cohort, it may be advisable to account for the imprecision of the measurement device which is used to estimate the average exposure for a group of workers in a group-level exposure assessment. In this vein, we can suppose an additional classical measurement error component, which is shared between and within workers for the for the first exposure period, as this period was characterised by a retrospective exposure reconstruction and the use of a single error prone exposure estimate for a group of workers and several years of exposure. For the second period, the imprecision of the measurement device that is used to obtain measurements for a group of workers in a prospective fashion will likely give rise to a component of classical measurement error that is shared between workers, but not for several years of the same worker. The combination of shared and unshared measurement error in the first and second exposure period can be expressed by model

$$\begin{array}{c} M_{10}:\{\begin{array}{c} X_{ij}^{1}\left(t\right)=\chi_{j}^{1}\cdot U_{ij}^{1}\left(t\right)\\ Z_{j}^{1}=\chi_{j}^{1}\cdot U_{j}^{1*}\\ X_{ij}^{2}\left(t\right)=L_{j}^{2}\left(t\right)\cdot U_{ij}^{2}\left(t\right)\\ Z_{j}^{2}\left(t\right)=L_{j}^{2}\left(t\right)\cdot U_{j}^{2*}\left(t\right)\\ Z_{ij}^{3}\left(t\right)=X_{ij}^{3}\left(t\right)\cdot U_{ij}^{3}\left(t\right). \end{array} \end{array}$$

where $\chi_{j}^{1}$ and $L_{j}^{2}\left(t\right)$ are latent intermediate variables. $\chi_{j}^{1}$ can be interpreted as the true average exposure value of group *j* in the retrospective exposure reconstruction in the first period. $L_{j}^{2}\left(t\right)$ represents the true average exposure value of group *j* at time *t* in the prospective group-level exposure assessment during the second period. In model $M_{10}$, we assume $E(U_{ij}^{1}\left(t\right)|\chi_{j}^{1})$, $E(U_{j}^{1*}|\chi_{j}^{1})$, $E(U_{ij}^{2}\left(t\right)|L_{j}^{2}\left(t\right))$, $E(U_{j}^{2*}\left(t\right)|L_{j}^{2}\left(t\right))$ and $E(U_{ij}^{3}\left(t\right)|X_{ij}^{3}\left(t\right))$ all equal to one. Moreover, $\text{log}\left(U_{j}^{1*}\right)∼N\left(-\frac{\sigma_{1*}^{2}}{2},\sigma_{1*}^{2}\right)$ and $\text{log}\left(U_{j}^{2*}\left(t\right)\right)∼N\left(-\frac{\sigma_{2*}^{2}}{2},\sigma_{2*}^{2}\right)$. The error term $U_{j}^{1*}$ is supposed to be shared both between and within subjects and therefore only depends on group *j*. The error term $U_{j}^{2*}\left(t\right)$, on the other hand, is shared between, but not within subjects and therefore depends on group *j* and time *t* but not on worker *i*.

#### Accounting for individual worker practices in the periods of group-level exposure estimation

Several authors have pointed out that workers can receive systematically higher or lower exposure values than the exposure level that is measured for their job category, although they work in the same environment and perform basically the same tasks [17, 18]. For instance, a comparison between a prospective method of group-level exposure assessment and individual exposure assessment in the French cohort of uranium miners revealed that individual cumulative radon exposure was substantially underestimated for some workers but not for others when exposure was assessed at the group-level [22]. A possible explanation for this finding is that some of the workers sought relief from the strong airstream produced by a ventilation system in their break hours, thereby exposing themselves to very high radon concentrations. The ventilation system was installed in the mines as a measure of radiation protection and the access to areas where the airflow was too weak was formally forbidden. Miners who infringed this rule received systematically higher true exposure values than their estimated exposure level for all years of exposure, which were characterised by a method of group-level exposure assessment. To account for the effect of these individual worker characteristics and worker practices, it seems adequate to model exposure uncertainty as a combination of a component of unshared Berkson error and a component of Berkson error shared for several years of a worker when a method of group-based exposure assessment is used, expressed by model

$$\begin{array}{c} M_{11}:\{\begin{array}{c} X_{ij}^{1}\left(t\right)=Z_{j}^{1}\left(t\right)\cdot U_{ij}^{1*}\left(t\right)\cdot U_{i}^{1*}\\ X_{ij}^{2}\left(t\right)=Z_{j}^{2}\left(t\right)\cdot U_{ij}^{2*}\left(t\right)\cdot U_{i}^{2*}\\ Z_{ij}^{3}\left(t\right)=X_{ij}^{3}\left(t\right)\cdot U_{ij}^{3}\left(t\right). \end{array} \end{array}$$

In this model, we assume $\text{log}\left(U_{i}^{1*}\right)∼N\left(-\frac{\sigma_{1*}^{2}}{2},\sigma_{1*}^{2}\right)$ and $\text{log}\left(U_{i}^{2*}\right)∼N\left(-\frac{\sigma_{2*}^{2}}{2},\sigma_{2*}^{2}\right)$ with $E(U_{i}^{1*}|Z_{j}^{1})=1$ and $E(U_{i}^{2*}|Z_{j}^{2}\left(t\right))=1$.

## Simulation studies

### Motivating example

To mimic the exposure conditions of a “true” occupational cohort, we used information on annual radon exposure of the French cohort of uranium miners [23, 24] as basis for all simulations. The study design was approved of by the French Commission Nationale de l’Information et des Libertés (CNIL) and the data underlying the findings of this study is restricted by this commission. Radon is a noble and radioactive gas, resulting from the decay of uranium 238. As radon is considered to be the second most important cause of lung cancer after smoking, the main outcome of interest when it comes to radon exposure is lung cancer mortality. Estimated excess relative risk coefficients per 100 working level months (WLM) vary between 0.8 and 4.2 in occupational cohorts of miners [25] when risk estimation is not corrected for measurement error. Note that Excess relative risk (ERR) is related to relative risk (RR) by the relation *RR* = 1 + *ERR*. Radon exposure in cohorts of underground miners is classically expressed in working level months with one working level month approximately equal to 6.3 × 10^5^Bq h m^−3^.

The French cohort of uranium miners consists of 5086 uranium miners, who present an average follow-up of 35 years between 1945 and 2007 and an average duration of employment of 17 years between 1945 and 1999. The radon exposure data of this cohort reflect conditions which can be seen as typical for an occupational cohort with varying methods of exposure assessment, depending on the period of exposure. For the earliest period of mining (1945-1955), there was no systematic radon exposure monitoring in the mines and exposure values had to be reconstructed retrospectively by a group of experts. The second exposure period (1956-1982) was characterised by a method of group-based exposure monitoring, where information gathered through ambient measurements at work sites was used to estimate the individual exposure of each miner in a prospective fashion. Finally, for the latest period of exposure (1983-1999), radon exposure was assessed individually and prospectively via personal dosimetry. At the same time, starting in 1955, improvements in radiation protection of the workers led to a sharp exposure reduction between 1955 and 1956, which was subsequently followed by a continual decrease in annual radon exposure until the last mine closed in France in 1999. The average annual radon exposure value for exposed miners was 28.28 WLM in 1955 and 0.14 WLM in 1999.

Despite evidence of multiple sources of shared uncertainty in radon exposure in cohorts of underground miners, attempts to model measurement error in these cohorts so far mainly relied on the hypothesis that all exposure uncertainty can be described by unshared multiplicative measurement error [7, 8, 26–28]. In the French cohort of uranium miners, an attenuation of the exposure response curve for cumulative exposure values exceeding 100 WLM has been observed [26]. This shape of the exposure response curve, which has been observed in many other occupational cohorts [3, 4], persists after unshared measurement error is accounted for. Stram et al. (1999) [1] observe a similar phenomenon when analysing lung cancer mortality in the Colorado Plateau Uranium Miners cohort, where the risk for radon exposures received at a high-dose rate is estimated to be smaller than the risk estimated for radon exposures received at a low dose-rate. The authors refer to this phenomenon, which is commonly observed in cohorts of uranium miners, as the inverse dose-rate effect. When reanalysing lung cancer mortality in the cohort with revised exposure estimates based on a model-based imputation scheme, the authors find that the inverse dose-rate effect is greatly diminished. In this context, it is important to ascertain whether the attenuation of the exposure-response relationship observed in the French cohort of uranium miners could be due to components of shared exposure uncertainty that are not accounted for.

### Simulation study 1: The impact of shared and unshared measurement error on risk estimation

Based on our motivating example, we performed a series of simulations to assess the impact of shared and unshared exposure uncertainty components on risk estimation in proportional hazard models when statistical inference is not corrected for measurement error.

#### Models used for data generation

To generate failure times, we considered two alternative proportional hazards models $D_{1}$ and $D_{2}$ to describe the association between instantaneous hazard rate of death by lung cancer of miner *i* at age *t*, *h*_*i*_(*t*) and his cumulative radon exposure. Both disease models specify *h*_*i*_(*t*) as a function of cumulative radon exposure in 100 WLM, $X_{i}^{\text{cum}}\left(t\right)$ of worker *i* until time *t* and the baseline hazard *h*_0_(*t*) of lung cancer mortality at age *t*. They are given by

$$\begin{array}{c} D_{1}:h_{i}\left(t\right)=h_{0}\left(t\right)\left(1+\beta X_{i}^{\text{cum}}\left(t\right)\right) \end{array}$$

and

$$\begin{array}{c} D_{2}:h_{i}\left(t\right)=h_{0}\left(t\right)\exp\left(\beta X_{i}^{\text{cum}}\left(t\right)\right). \end{array}$$

$D_{1}$ represents an excess hazard ratio (EHR) model, which is commonly used to describe the association between cancer mortality and exposure to radon and to other sources of ionising radiation. $D_{2}$, on the other hand, is the more classical form of the Cox proportional hazards model. Cumulative radon exposure $X_{i}^{\text{cum}}\left(t\right)$ is a time-varying variable as it represents the sum over all annual exposure values that worker *i* in group *j* received before time *t*: $X_{i}^{\text{cum}}\left(t\right)=\sum_{u\leq t}X_{ij}\left(u\right)$.

We considered different measurement models to describe the association between true *X*_*ij*_(*t*) and observed exposure *Z*_*ij*_(*t*) of worker *i* at time *t* belonging to group *j*. We used models $M_{1}$ to $M_{8}$ presented earlier, which assume the same type of error for all exposure periods and specify Berkson and classical measurement error that is either unshared, shared between subjects, shared within subjects or shared both within and between subjects.

#### Data generation

To create unshared exposure uncertainty, measurement errors *U*_*ij*_(*t*) were sampled independently from a log-normal distribution for each miner *i* at every time *t*. For components of exposure uncertainty shared within workers, we generated only one log-normally distributed error *U*_*i*_ for each worker i, which was then used for all times *t* at which this worker was exposed to radon. Conversely, for exposure uncertainty shared between workers, we generated one log-normally distributed error term, *U*_*j*_(*t*) at each time *t*, which was affected to all workers *i* in group *j*. Finally, for an error component, that was both shared between and within workers, i.e., at the same time among a group of workers and for several years of the same worker, we generated one log-normally distributed error *U*_*j*_ for each group of workers, which was applied for all times at which a worker belonging to group *j* was exposed. When generating exposure data with Berkson error, we made the assumption that observed exposure *Z*_*ij*_(*t*) was equal to the observed exposure values in the French cohort of uranium miners and multiplied *Z*_*ij*_(*t*) by an error term to obtain true exposure *X*_*ij*_(*t*). Conversely, when generating exposure data with classical measurement error, we made the assumption that true exposure *X*_*ij*_(*t*) was equal to the observed exposure values in the cohort and multiplied *X*_*ij*_(*t*) by an error term to obtain observed exposure *Z*_*ij*_(*t*). Since the French cohort of uranium miners did not present a natural partition into groups of workers, we created homogeneous groups of workers via a hierarchical ascendant clustering algorithm after a multiple factor analysis based on some covariates concerning job characteristics: principal type of mine, principal location and principal type of job.

We adapted a method proposed by Henry (2014) [29] to generate failure times for a proportional hazards model with time-dependent covariates. To generate survival times, we used a piecewise constant model to specify baseline hazard *h*_0_(*t*) in both the EHR and the Cox proportional hazards model. All data generation was done in R (version 3.3.1).

We chose a true risk coefficient of *β* = 5 for the EHR model, which is in the same order of magnitude as the risk coefficient estimated in the French cohort of uranium miners when restricting analyses to exposure periods characterised by a prospective method of exposure assessment [24, 26]. In order to achieve a comparable strength of the association between radon exposure and lung cancer mortality for the Cox model and the EHR model, we chose a risk coefficient of *β* = 2 for the Cox model. For the sake of completeness, we included results for the EHR model with *β* = 2 and for the Cox model with *β* = 5 in Table 4 and Table 5 in S2 File.

We compared the impact of large and moderate measurement error, corresponding to values for the variance of measurement error of $\sigma_{\epsilon }^{2}=0.8$ and $\sigma_{\epsilon }^{2}=0.1$, respectively. These values were chosen in accordance with the characterisation of exposure uncertainty in the French cohort of uranium miners [27, 30]. While a measurement error variance of $\sigma_{\epsilon }^{2}=0.1$ is likely to occur in the more recent exposure periods of an occupational cohort, a measurement error variance of $\sigma_{\epsilon }^{2}=0.8$ may be assumed for the earliest exposure years for which exposure values are commonly reconstructed in a retrospective fashion.

The combination of all possibilities for the disease model, the measurement model, for the value of the true risk coefficient and for the measurement error variance resulted in 2 × 8 × 2 × 2 = 64 distinct simulation scenarios. Additionally, we compared the results with risk estimates when radon exposure was observed without measurement error, i.e. under measurement model $M_{0}$, resulting in 68 simulation scenarios in total.

#### Assessment of risk estimates

For each scenario, inference was based on 100 simulated data sets. We conducted inference for disease model $D_{1}$ for data sets that were generated according to the EHR model. Likewise, we conducted inference for the disease model $D_{2}$ for data sets that were generated according to the Cox model. Observed exposure *Z*_*ij*_(*t*) was treated as an error-free surrogate of true exposure *X*_*ij*_(*t*) in the analysis of the association between exposure and disease outcome, i.e. measurement error was not accounted for in risk estimation. We used a Metropolis-Hastings algorithm developed and tested in Python version 2.7 for Bayesian inference. We chose a centred normal prior distribution with a large variance (1000) for the risk coefficient *β*, which was truncated to guarantee the positivity of *h*_*i*_(*t*) in the EHR model. After checking convergence, inference was based on 20.000 iterations after an initial burn-in phase of 10.000 iterations (thin = 1).

For each scenario, we estimated:

1. An overall 95% credible interval (*CI*_95%_), which was obtained by combining the chains of the 100 replicates for each scenario and by determining the 2.5 and 97.5 quantiles of the corresponding pooled chain.
2. The relative bias of a Bayesian point estimator $\hat{\beta }$, given by $\frac{\left(\hat{\beta }-\beta \right)}{\beta }$, where *β* is the risk coefficient which served to generate the data. We used the posterior median as Bayesian point estimate for the risk coefficient $\hat{\beta }$.
3. The coverage rate of 95% credible intervals, which was calculated by counting the proportion of the 100 replicates for which the 95% credible interval included the true value of the coefficient *β*
4. The statistical power, which was estimated by counting the proportion of replicates for which the 95% credible interval for *β* excluded 0.

### Simulation study 2: The effects of measurement error characteristics on the shape of the exposure-response curve

We performed a second series of simulations to assess the effects of different error structures on the observed shape of the exposure-response curve when risk estimation is not corrected for measurement error.

#### Models used for data generation

We used models $D_{1}$ (EHR model) and $D_{2}$ (Cox model), described earlier to generate mortality data as a function of true cumulative radon exposure $X_{i}^{\text{cum}}\left(t\right)$.

We considered model $M_{0}$ with no measurement error, model $M_{1}$ describing unshared Berkson error for all exposure periods and the more complex and more plausible models $M_{9},M_{10}$ and $M_{11}$. $M_{9}$ only describes unshared error, thereby accounting for differences in type and magnitude of error occurring in the three exposure periods, while $M_{10}$ and $M_{11}$ also allow for shared components of exposure uncertainty due to the imprecision of the measurement device and individual worker practices, respectively.

#### Data generation

For model $M_{10}$, the latent intermediate variables $\chi_{j}^{1}$ and $L_{j}^{2}\left(t\right)$ were set to the exposure values observed in the French cohort of uranium miners and both observed exposure *Z*_*ij*_(*t*) and true exposure *X*_*ij*_(*t*) were obtained by multiplying these intermediate variables with shared and unshared measurement error, respectively. For all other models, Berkson and classical measurement error were generated according to the strategy described earlier.

We chose *β* = 2 for the Cox model and *β* = 5 for the EHR model. Results for the EHR model with *β* = 2 and for the Cox model with *β* = 5 can be found in S1 and S2 Figs and Table 6 of S2 File.

In order to be able to assess the impact of the structure of measurement error, rather than the magnitude of error, we tried to keep the total magnitude of error constant for all models. Therefore, we generated data according to model $M_{9}$ with $\sigma_{1}^{2}=0.8$, $\sigma_{2}^{2}=0.15$ and $\sigma_{3}^{2}=0.01$, following Allodji et al. [27, 30]. To obtain a global variance of log-transformed errors comparable to this scenario, we set *σ*^2^ equal to 0.2 in the homoscedastic Berkson error model $M_{1}$. In accordance with the characterisation of exposure uncertainty made by Allodji et al. [30], the variance parameters in model $M_{10}$ accounting for the imprecision on the measurement device as shared source of uncertainty, were chosen as $\sigma_{1}^{2}=0.09,\sigma_{2}^{2}=0.03$ and $\sigma_{3}^{2}=0.01,\sigma_{1*}^{2}=0.81$ and $\sigma_{2*}^{2}=0.12$. For the variance parameters in model $M_{11}$, accounting for individual worker practices as shared source of uncertainty, we chose the same values for the variance parameters as for model $M_{10}$. In doing so, we are able to compare the effects of shared exposure uncertainty due to the imprecision on the measurement device in a group-level exposure estimation and due to individual worker practices for a given error variance.

#### Assessing the shape of the exposure-response curve

We conducted statistical inference for both models $D_{1}$ and $D_{2}$ for all data sets, regardless of the disease model that was used for data generation, to study the effects of different error structures on disease model choice when inference is not accounted for measurement error. The Deviance Information Criterion (DIC) was used to compare the competing disease models. The DIC is a Bayesian model selection criterion, which can be seen as a penalised likelihood criterion evaluating the trade-off between goodness of fit and model complexity with smaller DIC values indicating a better fit to the data.

To investigate the possibility of measurement error to induce a non-linear exposure-response relationship, we estimated parameter values in an EHR ($D_{3}$) and a Cox model ($D_{4}$) based on natural cubic splines. In these models, we chose interior knots at the 20th, 40th, 60th and 80th percentile of the exposure distribution of cases, i.e. miners who died of lung cancer in our simulation study.

While these disease models allow for a graphical evaluation of the impact of different measurement error characteristics, the parameter estimates in these models are not easily interpretable. Consequently, we also fitted continuous piecewise-linear models with a breakpoint at 100 WLM to be able to complement the results of model $D_{3}$ and $D_{4}$ with slope estimates for high and low exposure values under the different error structures. $D_{3}$ and $D_{5}$ were estimated for data sets that were generated according to the linear EHR model $D_{1}$ to assess the effect of the different measurement error characteristics when the EHR model was the true disease model. Similarly, $D_{4}$ and $D_{6}$ were fitted for data sets that were generated according to the linear Cox model $D_{2}$. All statistical inference was based on the assumption that observed exposure $Z_{ij}^{q}\left(t\right)$ was a perfect surrogate of true exposure $X_{ij}^{q}\left(t\right)$. The natural cubic spline basis was constructed in R and Bayesian inference via a Metropolis-Hastings algorithm was conducted in Python. Inference was based on 20.000 iterations after an initial burn-in of 10.000 iterations (thin = 1).

## Results

### The impact of shared and unshared measurement error on risk estimation

Table 1 shows risk estimates and overall 95% credible intervals in the Cox proportional hazards model. Exposure uncertainty shared within workers created more relative bias in risk estimates and smaller coverage rates than exposure uncertainty shared between workers. The relative bias of small measurement error of both Berkson and classical nature, for instance, was more than twice as large when this error was shared within workers rather than between workers. In general, the impact of unshared uncertainty and uncertainty shared between workers was comparable. Error components, which were both shared between and within individuals produced about as much bias as error components that were only shared within individuals.

**Table 1:** Average posterior median ($\hat{\beta }$), overall 95% credible intervals (CI_95%_), relative bias and coverage rate for 100 data sets generated according to the Cox model $D_{2}$, a measurement model among $M_{0}$ to $M_{8}$ and a true risk coefficient of *β* = 2 per 100 WLM.

Model	Type of sharing	Type of error	Error variance	$\hat{\beta }$	CI_95%_	Relative bias	Coverage rate
$M_{1}$	unshared	Berkson	0.1	1.81	[1.64; 1.99]	-0.10	0.10
			0.8	1.25	[0.97; 1.49]	-0.38	0.00
$M_{2}$		classical	0.1	1.75	[1.55; 1.93]	-0.13	0.02
			0.8	0.83	[0.45;1.21]	-0.59	0.00
$M_{3}$	between	Berkson	0.1	1.82	[1.62; 2.01]	-0.09	0.22
			0.8	1.25	[1.03; 1.47]	-0.38	0.00
$M_{4}$		classical	0.1	1.75	[1.53; 1.94]	-0.13	0.05
			0.8	0.80	[0.44; 1.16]	-0.60	0.00
$M_{5}$	within	Berkson	0.1	1.45	[1.12, 1.69]	-0.28	0.00
			0.8	0.76	[0.54; 0.97]	-0.62	0.00
$M_{6}$		classical	0.1	1.33	[1.04; 1.58]	-0.34	0.00
			0.8	0.39	[0.17; 0.62]	-0.81	0.00
$M_{7}$	both	Berkson	0.1	1.46	[1.11; 1.78]	-0.27	0.00
			0.8	0.77	[0.54; 1.00]	-0.62	0.00
$M_{8}$		classical	0.1	1.42	[1.04; 1.72]	-0.29	0.00
			0.8	0.49	[0.13; 0.86]	-0.76	0.00
$M_{0}$	none	none	0	1.96	[1.80; 2.13]	-0.02	0.95

Table 2 presents the same summary statistics concerning risk estimates as Table 1 but for failure times generated according to the EHR model. The relative bias introduced by measurement error in the EHR model was smaller than the bias introduced in the Cox model. For large measurement error in the EHR model, we observed the same pattern as for the Cox model where measurement errors shared within workers caused more relative bias and lower coverage rates than unshared measurement error or measurement error that was only shared between workers. For small measurement error, this tendency was less evident. In general, classical measurement caused more relative bias and smaller coverage rates than Berkson error and large measurement error caused more relative bias and smaller coverage rates than small measurement error, regardless of the disease model and regardless of whether exposure uncertainty was shared or unshared. When data were generated without exposure measurement error, the coverage rates of 95% credible intervals were very close to 95%.

**Table 2:** Average posterior median ($\hat{\beta }$), overall 95% credible intervals (CI_95%_), relative bias and coverage rate for 100 data sets generated according to the EHR model $D_{1}$, a measurement model among $M_{0}$ to $M_{8}$ and a true risk coefficient of *β* = 5 per 100 WLM.

Model	Type of sharing	Type of error	Error variance	$\hat{\beta }$	CI_95%_	Relative bias	Coverage rate
$M_{1}$	unshared	Berkson	0.1	4.87	[3.07; 7.68]	-0.03	0.93
			0.8	4.65	[2.89; 7.18]	-0.07	0.91
$M_{2}$		classical	0.1	4.88	[3.13;7.47]	-0.02	0.94
			0.8	4.34	[2.71; 6.70]	-0.13	0.78
$M_{3}$	between	Berkson	0.1	4.77	[3.14; 7.11]	-0.05	0.99
			0.8	4.69	[2.91; 7.31]	-0.06	0.93
$M_{4}$		classical	0.1	4.79	[3.04; 7.35]	-0.04	0.93
			0.8	4.44	[2.82; 6.72]	-0.11	0.85
$M_{5}$	within	Berkson	0.1	4.88	[3.13; 7.47]	-0.02	0.94
			0.8	3.98	[2.43; 6.23]	-0.20	0.73
$M_{6}$		classical	0.1	4.75	[3.01; 7.31]	-0.05	0.91
			0.8	3.03	[1.86; 4.71]	-0.39	0.13
$M_{7}$	both	Berkson	0.1	4.88	[3.11; 7.69]	-0.02	0.94
			0.8	3.86	[2.19; 6.59]	-0.23	0.55
$M_{8}$		classical	0.1	4.76	[2.94; 7.29]	-0.05	0.92
			0.8	3.15	[1.62; 5.25]	-0.37	0.25
$M_{0}$	none	none	0	4.90	[3.14; 7.45]	-0.02	0.96

The statistical power was estimated to be 100% for all scenarios, both for data generated according to the Cox model and according to the EHR model.

### The effects of measurement error characteristics on the shape of the exposure-response curve

**Fig 1:**
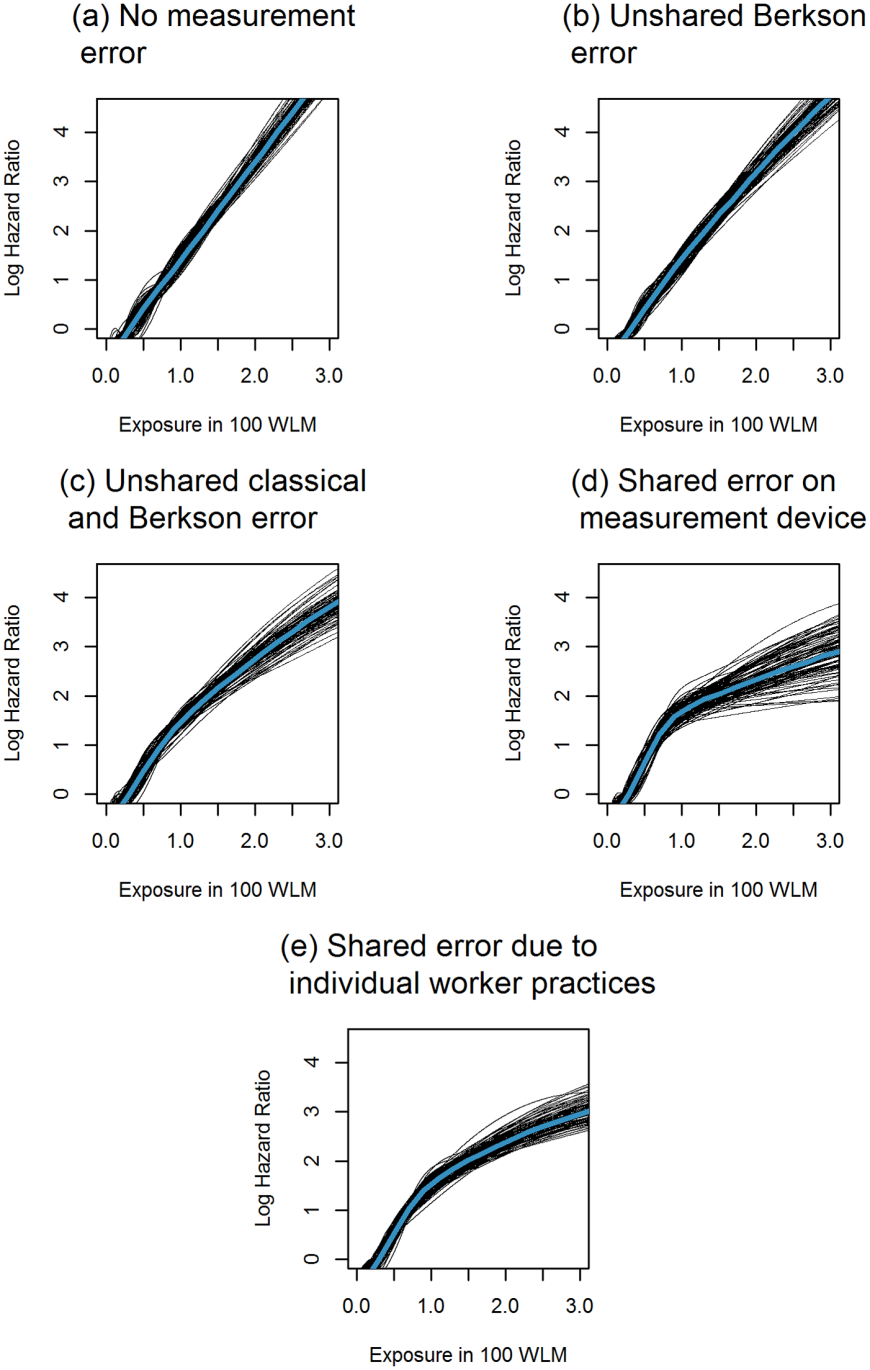
Estimated exposure-response curve when fitting the Cox model $D_{4}$ based on natural cubic splines when data are generated according to the Cox model $D_{2}$ with a risk coefficient of *β* = 2. (a) $M_{0}$, i.e., no measurement error (b) $M_{1}$, i.e., unshared and homoscedastic Berkson error, (c) $M_{9}$, i.e., unshared error of Berkson and classical type (d) $M_{10}$, i.e., heteroscedastic error with a shared classical component describing the imprecision of the measurement device and (e) $M_{11}$, i.e., heteroscedastic error with a shared Berkson component describing individual worker practices.

As can be seen in Fig 1, exposure-response curves for data generated according to the Cox model with no measurement error ($M_{0}$) or unshared and homoscedastic Berkson error ($M_{1}$) were close to linear on the log-scale. Heteroscedastic unshared error ($M_{9}$) appeared to create a slightly non-linear association. Indeed, Table 3 confirms that the slope for exposure under 100 WLM in this scenario is estimated to be more than twice as large as for exposure values over 100 WLM. Moreover, according to the DIC, the EHR model fitted the data better than the Cox model in 34% of cases when exposure data were generated following this unshared and heteroscedastic measurement model, even though the true disease model was the Cox model. For data generated according to the measurement models which incorporated shared sources of uncertainty ($M_{10}$ and $M_{11}$) the attenuation of the exposure-response curve at high exposure values was even more noteworthy. The slope estimates for low exposures in these scenarios were about six to eight times larger than the slope estimates for high exposures. Under these scenarios, DIC values indicated for all replicates that the EHR model fit the data better than the Cox model, although data were generated according to the Cox model. In the three scenarios using heteroscedastic measurement models ($M_{9},M_{10}$ and $M_{11}$), risk coefficients estimated in the piecewise-linear disease model $D_{6}$ were overestimated for low exposures and underestimated for high exposure values when data was generated according to the Cox model. Overall, we only observed a substantial attenuation of the exposure-response curve in the Cox model when the first exposure period was characterised by a mixture of unshared and shared measurement error, which was either shared within workers or both within and between workers.

**Table 3:** Comparison of risk estimates when data are generated according to different disease and measurement models. DIC_*EHR*_ < DIC_*Cox*_ gives the percentage of realisations for which the Deviance Information Criterion (DIC) was smaller for the Excess Hazard Ratio (EHR) model when the true model was the Cox model and vice versa for DIC_*Cox*_ < DIC_*EHR*_. The difference in DIC is calculated as difference between the EHR model and the Cox model.

Disease model	Model $M_{0}$	Model $M_{1}$	Model $M_{9}$	Model $M_{10}$	Model $M_{11}$
	No error	Unshared Berkson error	Unshared heteroscedastic Berkson and classical error	Heteroscedastic shared device	Heteroscedastic worker practices
**Data generated according to the Cox model ($D_{2}$) with *β* = 2**					
Risk estimate $\hat{\beta }$ in the linear Cox model ($D_{2}$)	1.97 [1.78; 2.16]	1.67 [1.50; 1.87]	1.23 [1.00; 1.42]	0.57 [0.21; 1.06]	0.77 [0.59; 0.98]
Risk estimates in the piecewise-linear Cox model ($D_{6}$)					
${\hat{\beta }}_{1}$ (under 100 WLM)	1.98 [1.57; 2.40]	2.08 [1.65; 2.49]	2.21 [1.78; 2.61]	2.50 [2.06; 2.91]	2.33 [1.93; 2.70]
${\hat{\beta }}_{2}$ (over 100 WLM)	1.96 [1.68; 2.26]	1.49 [1.22; 1.80]	0.92 [0.64; 1.18]	0.31 [0.06; 0.68]	0.40 [0.20; 0.63]
DIC_*EHR*_ < DIC_*Cox*_	0%	0%	34%	99%	100%
Difference in DIC	-216.08	-142.13	-15.17	169.16	107.24
**Data generated according to the EHR model ($D_{1}$) with *β* = 5**					
Risk estimate $\hat{\beta }$ in the in the linear EHR model ($D_{1}$)	4.90 [3.24; 7.62]	4.71 [3.08; 7.19]	4.44 [2.93; 6.81]	4.11 [2.26; 7.21]	4.07 [2.49; 6.28]
Risk estimates in the piecewise-linear EHR model ($D_{5}$)					
${\hat{\beta }}_{1}$ (under 100 WLM)	4.95 [2.83; 8.33]	4.81 [2.91; 7.67]	4.75 [2.79; 7.59]	5.58 [3.38; 9.16]	4.73 [2.77; 7.64]
${\hat{\beta }}_{2}$ (over 100 WLM)	5.14 [2.06; 9.17]	4.72 [2.05; 9.21]	4.16 [1.48; 7.71]	2.18 [0.27; 6.43]	3.09 [0.69; 6.40]
DIC_*Cox*_ < DIC_*EHR*_	0%	0%	0%	0%	0%
Difference in DIC	93.76	87.98	85.62	132.64	83.10

**Fig 2:**
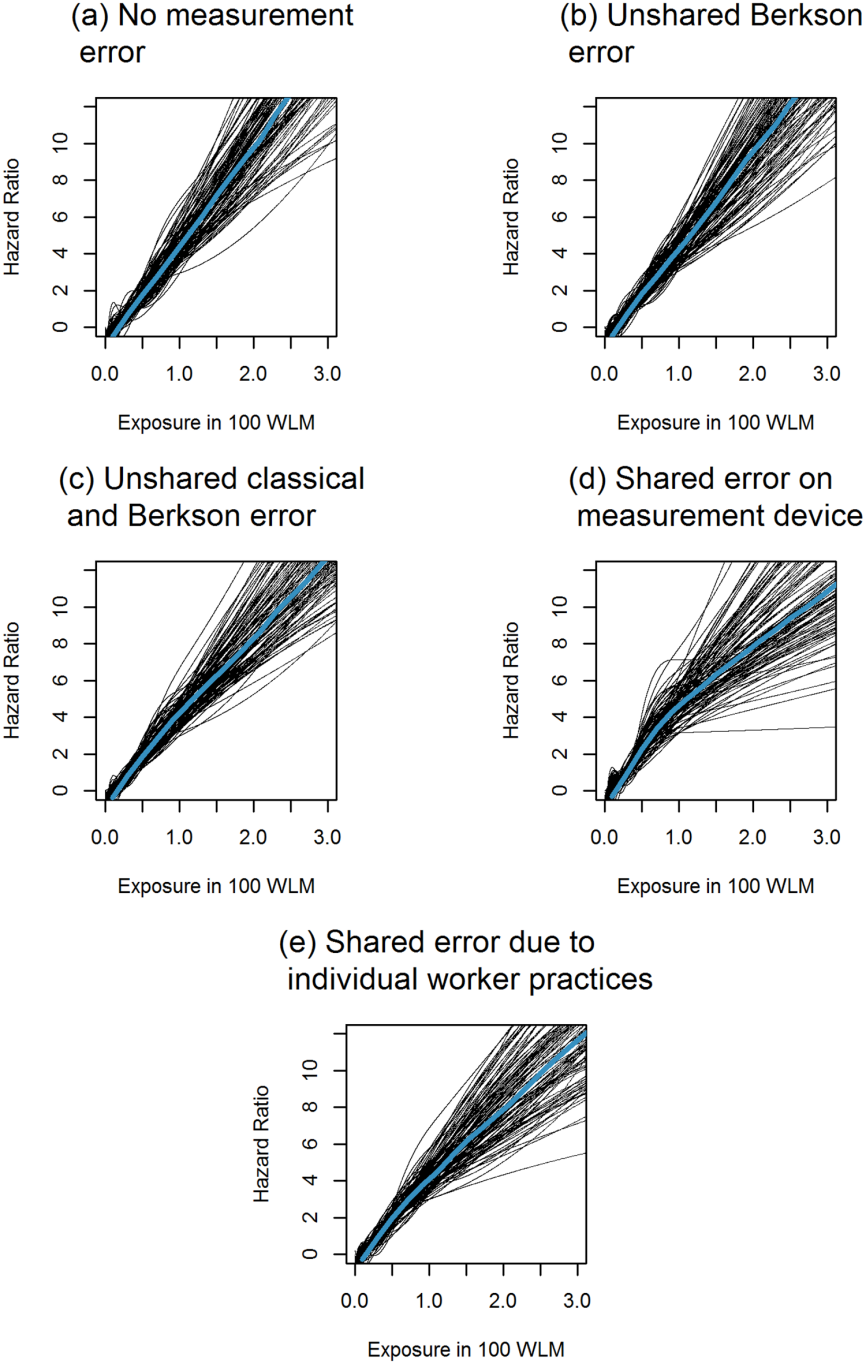
Estimated exposure-response curve when fitting the Excess Hazard Ratio (EHR) model $D_{3}$ based on natural cubic splines when data are generated according to the EHR model $D_{1}$ with a risk coefficient of *β* = 5. (a) $M_{0}$, i.e., no measurement error (b) $M_{1}$, i.e., unshared and homoscedastic Berkson error, (c) $M_{9}$, i.e., unshared error of Berkson and classical type (d) $M_{10}$, i.e., heteroscedastic error with a shared classical component describing the imprecision of the measurement device and (e) $M_{11}$, i.e., heteroscedastic error with a shared Berkson component describing individual worker practices.

Fig 2 suggests that the different patterns of shared or unshared measurement error did not produce any notable attenuation in exposure-response curves when mortality data were generated according to the EHR model. The risk estimates in the piecewise linear EHR model in Table 3 reveal that the risk for exposures under 100 WLM is estimated to be more than twice as large as the risk estimated for exposures exceeding 100 WLM when exposure data is contaminated with components of Berkson error which are shared for several years of the same worker ($M_{10}$). This measurement model is the only model for which the risk estimate for low exposures is estimated to be higher than the risk coefficient that was chosen to generate the data. For shared error components reflecting the imprecision of the measurement device ($M_{11}$), we also observe a higher risk estimate for exposures under 100 WLM than for exposures exceeding 100 WLM in the piecewise linear EHR model. However, in both cases, the credible intervals for the parameters in the piecewise linear model are very large and overlap. In contrast to the Cox model, DIC values always indicated the EHR to be the better fitting disease model when failure times were generated according to the EHR model, regardless of the measurement model.

## Discussion

In the present simulation study, we compared the effects of shared and unshared uncertainty in cumulative exposure in an occupational cohort study on risk estimation and on the shape of the exposure-response relationship in proportional hazards models. In general, exposure uncertainty shared within individuals (i.e., shared for several years of exposure for an individual) caused more bias in risk estimates and smaller coverage rates than unshared exposure uncertainty. In contrast to claims that uncertainty shared between individuals should have fundamentally different effects on parameter estimation than unshared exposure uncertainty [15, 16], we found that both error components resulted in comparable relative bias and coverage rates in risk estimation in proportional hazard models. In line with previous findings on the impact of measurement error, we found that classical measurement error had more impact on inference than Berkson error [2], regardless of the extent and type of sharing. While we chose a Bayesian approach to conduct statistical inference on risk estimates, frequentist likelihood-based inference yields the same results when it comes to the bias introduced by different components of measurement error (results not shown) as we assumed flat prior distributions. In line with this argument, concerning the relative bias in risk estimates in the presence of large and moderate unshared Berkson error, we observed values that were consistent with the results of Bender et al. (2005) and Küchenhoff et al. (2007), who studied the effect of unshared additive and multiplicative Berkson error on frequentist inference conducted for the Cox model. When studying the association between a disease outcome and cumulative exposure, we found that measurement error shared within individuals had more impact on risk estimation than measurement error shared between individuals. This finding is in accordance with the general principle that the impact of measurement error strongly depends on the variance of exposure and the variance of measurement error [2, 31]. In order to obtain cumulative exposure values in an occupational cohort study, the annual exposure values for a worker have to be summed and an error term shared within workers will be repeated for several exposure values in that sum. As the variance of the sum of positively correlated variables is greater than the sum of their variances, this summing will increase the measurement error variance in cumulative exposure. Uncertainty components shared between workers are unlikely to have a similar effect, because exposure values are summed within workers and not between workers. While it is therefore not surprising that error components shared within workers have more impact on statistical inference than components shared between workers when the main risk factor of interest is cumulative exposure, this result has important implications for the analysis of occupational cohort studies. In particular, this finding casts doubt on the common practice to model measurement error occurring in the exposure history of a worker on the sum of these values [4, 5, 32], instead of modelling on their natural level of occurrence, namely on the monthly or annual exposure values. In making this simplifying assumption, one may mistakenly model an error component that is shared for several years of a worker as an unshared error component. Our results suggest that may yield highly misleading results. We found that the impact of error components that were shared both within and between workers was comparable to the impact of error components that were shared between workers. However, it is likely that an error component that is shared for all members of a cohort could have even larger effects on statistical inference than an error component that is only shared for a sub-group of workers. Moreover, it is important to note that our findings are sensitive to the number of exposure values that are summed for a worker. In this study, we calculated cumulative exposure for a worker by summing his annual exposure values. In an occupational cohort study in which monthly exposure values are available for each worker, an error component that is shared within workers is likely to have even more impact on inference than in our study.

In accordance with the results obtained by Steenland et al. [5], we only observed a mild attenuation of the exposure-response curve in the Cox model when assuming a structure of unshared error in which the magnitude of error and the magnitude of exposure was greatest for the earliest years of exposure, which are often characterised by retrospective exposure reconstruction. However, in an occupational cohort, it seems more plausible to assume shared error components due to the imprecision of the measurement device and individual worker practices when exposure values are retrospectively reconstructed. Under these assumptions, we found a considerable attenuation in the exposure-response relationship for high exposure values when data were generated according to the Cox model. Attenuations of the exposure-response curve at high exposure values may pose serious challenges in risk modelling in occupational cohort studies. Indeed, if this attenuation reflects the association between true exposure and the outcome and a linear model is chosen, it may cause a severe underestimation of risk for workers with low exposures. On the other hand, if the association between true exposure and the outcome is linear and the observed distortion of the exposure-response relationship is caused by measurement error, fitting a non-linear or a piecewise-linear model can lead to an overestimation of the risk coefficient for workers with low exposures. To support radiation protection, researchers are particularly interested in the low exposure range, because exposure levels of workers are currently much lower than in the past. Moreover, these exposure values are comparable to exposures received by the general population. Ignoring the cause of an observed distortion of the exposure-response curve may therefore seriously limit the extrapolability of risk estimates obtained in occupational studies to the general population.

In accordance with previous findings concerning the relative importance of measurement error in linear and log-linear models [32], we found that distortions in the exposure-response relationship were more severe when data were generated according to the Cox model, rather than according to the EHR model. Moreover, when failure times were generated according to the Cox model and observed exposure values were contaminated with shared and unshared error, DIC values identified the EHR model as the model that best fitted the data. On the one hand, the robustness to measurement error makes the EHR model, which is often considered the best model to describe the effects of ionising radiation on mortality, attractive for risk modelling in epidemiological studies. On the other hand, this finding casts doubt on the possibility to identify a “true model” to describe the exposure-risk relationship when risk estimates are not corrected for all sources of exposure uncertainty.

Concerning the impact of measurement error in radon exposure in the French cohort of uranium miners, our findings strengthen the hypothesis that the observed attenuation of the exposure response relationship might be caused by components of shared measurement error, as these components are likely to have occurred in the first exposure period of the cohort. Moreover, they call into question the results of previous studies accounting for exposure uncertainty, as these studies relied on the hypothesis that all exposure uncertainty occurring in this cohort could be described by unshared measurement error [26–28].

More generally, the results of the present study underline the importance of making a careful characterisation of shared and unshared exposure uncertainty in observational studies if the aim is to account for its potential impacts on statistical inference. In particular, one should be aware of the distortions of the exposure response relationship that may be induced by different degrees of precision and varying amounts of sharing. To obtain corrected risk estimates, it is important to use statistical methods that allow for complex patterns of shared and unshared measurement error. As measurement error shared within individuals appears to have more impact on risk estimation than unshared error components or error components shared between individuals, it is important to correctly specify these error components as such and to account for the fact that the type of exposure uncertainty may vary over time. As far as we know, there is currently no possibility to use classical methods, such as regression calibration or simulation extrapolation to handle these complex patterns of measurement error. Recently, a number of methods have been proposed to account for shared error components through the integration of multiple realizations of exposure estimates in risk estimation [13–15]. In our view, the Bayesian hierarchical approach is another promising framework in this context [26, 33]. It is arguably the most flexible approach to account for exposure uncertainty and corrected parameter estimates can be obtained by Markov Chain Monte Carlo sampling. Additionally, the integration of prior knowledge on unknown parameters available from previous studies or in the form of expert knowledge can lead to more precise risk estimates and help to avoid overfitting, thereby increasing the replicability of findings.

The results of the present study may not only provide new insights in the interpretation and the discussion of analyses conducted on current occupational cohorts, but also for the design of future epidemiological studies. Methods of individual exposure assessment are becoming more accessible than ever with technical advances that facilitate the collection of exposure data. It is often argued that exposure uncertainty in group-level exposure estimation will not bias risk estimates, by combining the two simplifying assumptions that a group-level exposure estimation leads to Berkson error and that Berkson error does not bias risk estimates [5, 6, 34]. The results of the present study suggest that both of these simplifying assumptions do not hold in general and that shared components of Berkson error can even lead to a substantial distortion of the exposure-response relationship in the Cox model. In our view, a method of individual exposure assessment should be preferred over a method of group-level exposure estimation to avoid uncertainty components shared within workers and between workers, which may arise in a method of group-level exposure estimation because of the imprecision of the measurement device and individual worker practices.

## Supporting information

S1 FigEstimated exposure-response curve when fitting the Cox model $D_{4}$ based on natural cubic splines when data are generated according to the Cox model $D_{2}$ with a risk coefficient of *β* = 2.(a) $M_{0}$, i.e., no measurement error (b) $M_{1}$, i.e., unshared and homoscedastic Berkson error, (c) $M_{9}$, i.e., unshared error of Berkson and classical type (d) $M_{10}$, i.e., heteroscedastic error with a shared classical component describing the imprecision of the measurement device and (e) $M_{11}$, i.e., heteroscedastic error with a shared Berkson component describing individual worker practices.(TIFF)

S2 FigEstimated exposure-response curve when fitting the Excess Hazard Ratio (EHR) model $D_{3}$ based on natural cubic splines when data are generated according to the EHR model $D_{1}$ with a risk coefficient of *β* = 5.(a) $M_{0}$, i.e., no measurement error (b) $M_{1}$, i.e., unshared and homoscedastic Berkson error, (c) $M_{9}$, i.e., unshared error of Berkson and classical type (d) $M_{10}$, i.e., heteroscedastic error with a shared classical component describing the imprecision of the measurement device and (e) $M_{11}$, i.e., heteroscedastic error with a shared Berkson component describing individual worker practices.(TIFF)

S3 FigEstimated exposure-response curve when fitting the Excess Hazard Ratio (EHR) model $D_{3}$ based on natural cubic splines when data are generated according to the EHR model $D_{1}$ with a risk coefficient of *β* = 5 assuming additive measurement error.(a) $M_{0}$, i.e., no measurement error (b) $M_{1}$, i.e., unshared and homoscedastic Berkson error, (c) $M_{9}$, i.e., unshared error of Berkson and classical type (d) $M_{10}$, i.e., heteroscedastic error with a shared classical component describing the imprecision of the measurement device and (e) $M_{11}$, i.e., heteroscedastic error with a shared Berkson component describing individual worker practices.(TIFF)

S1 FileA more detailed presentation of measurement models $M_{5}$, $M_{6}$, $M_{7}$ and $M_{8}$.(PDF)

S2 File Complementary results. (PDF)
