# Supplementary figures and images for: Shared and unshared exposure measurement error in occupational cohort studies and their effects on statistical inference in proportional hazards models
Source: PLoS One. 2018 Feb 6;13(2):e0190792. doi: 10.1371/journal.pone.0190792 (PMC5800563; doi:10.1371/journal.pone.0190792)

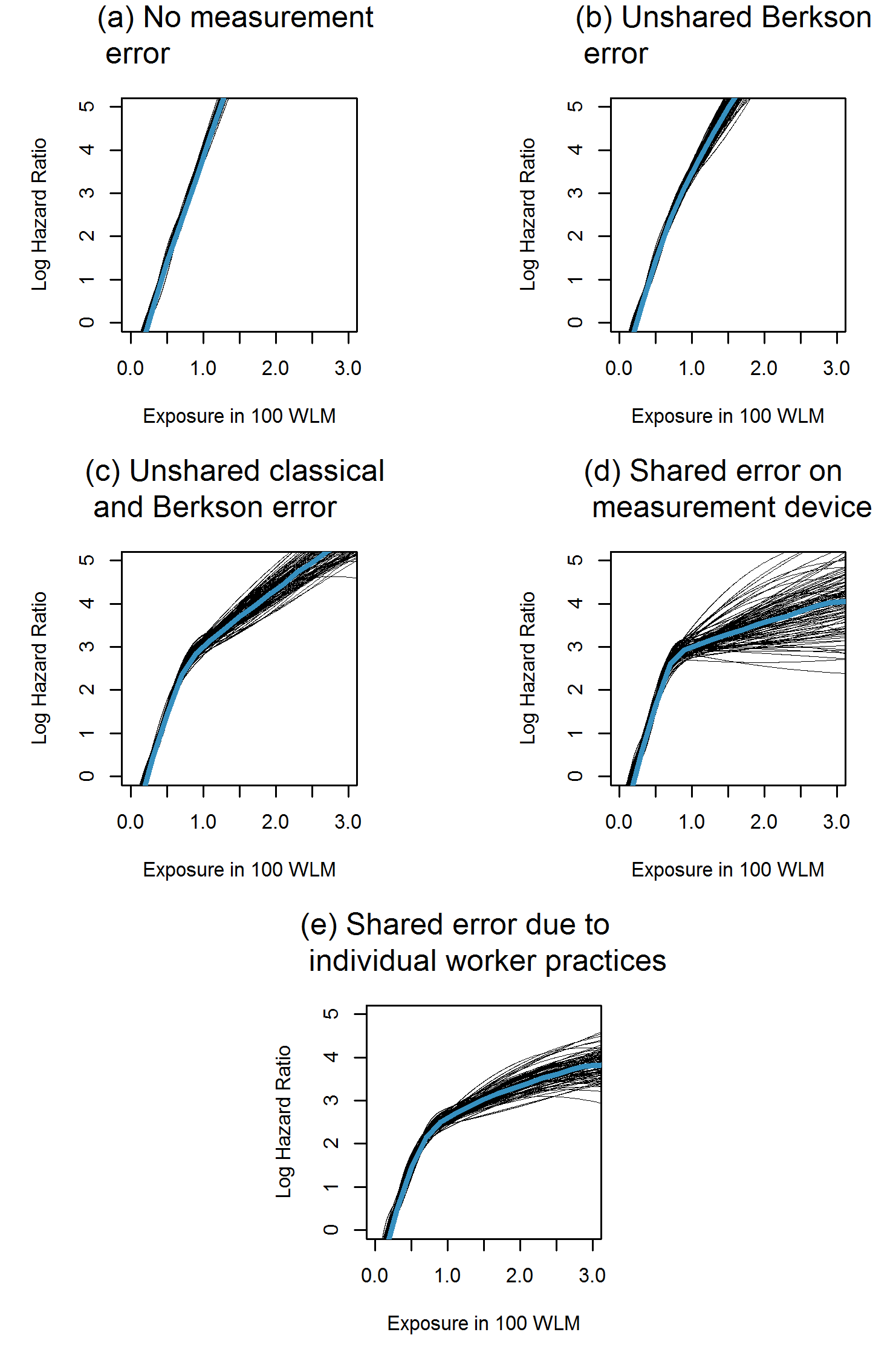

Supplement: S1 Fig — (a) M0, i.e., no measurement error (b) M1, i.e., unshared and homoscedastic Berkson error, (c) M9, i.e., unshared error of Berkson and classical type (d) M10, i.e., heteroscedastic error with a shared classical component describing the imprecision of the measurement device and (e) M11, i.e., heteroscedastic error with a shared Berkson component describing individual worker practices. (TIFF) [file pone.0190792.s001.tiff]

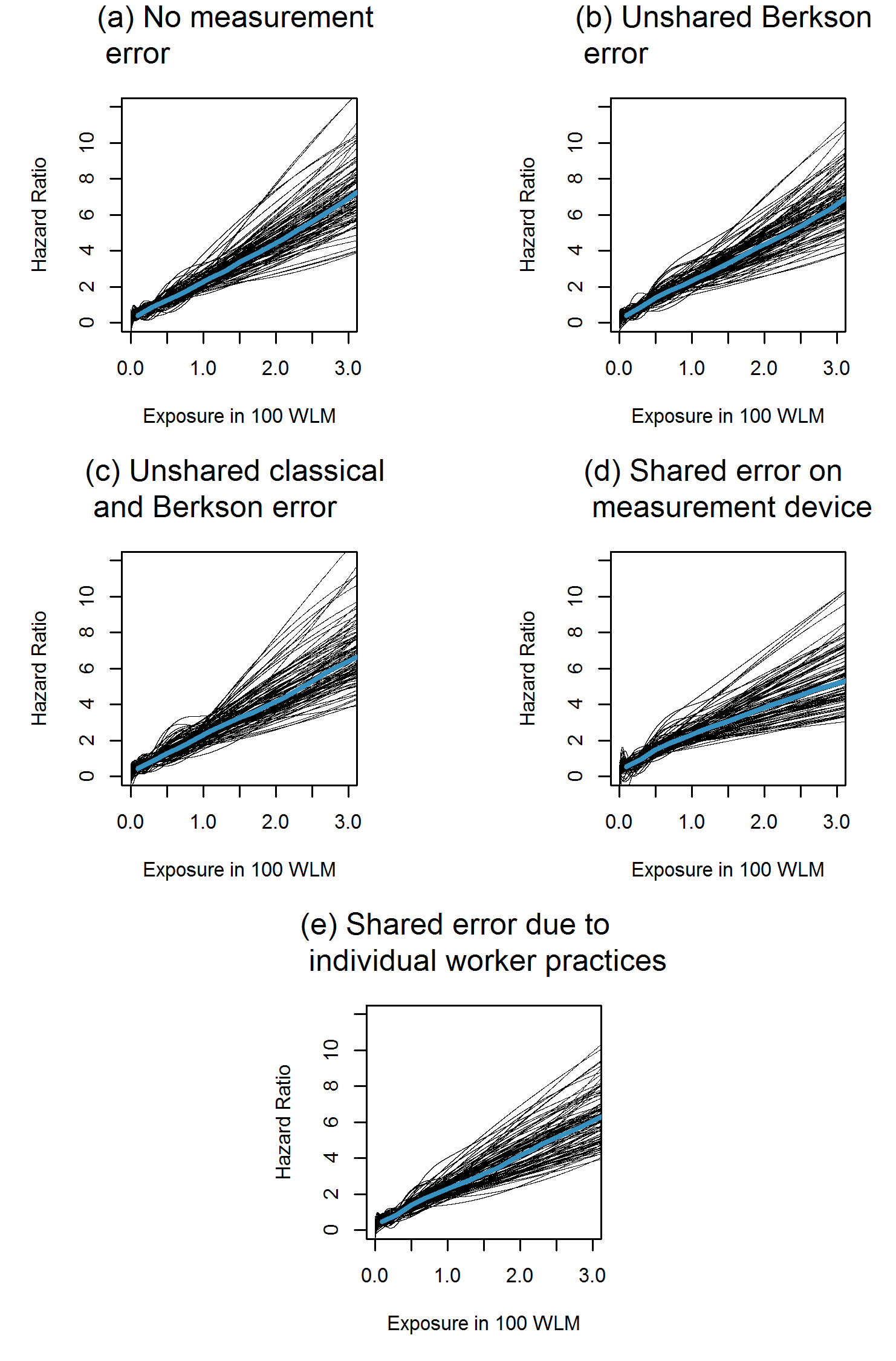

Supplement: S2 Fig — (a) M0, i.e., no measurement error (b) M1, i.e., unshared and homoscedastic Berkson error, (c) M9, i.e., unshared error of Berkson and classical type (d) M10, i.e., heteroscedastic error with a shared classical component describing the imprecision of the measurement device and (e) M11, i.e., heteroscedastic error with a shared Berkson component describing individual worker practices. (TIFF) [file pone.0190792.s002.tiff]

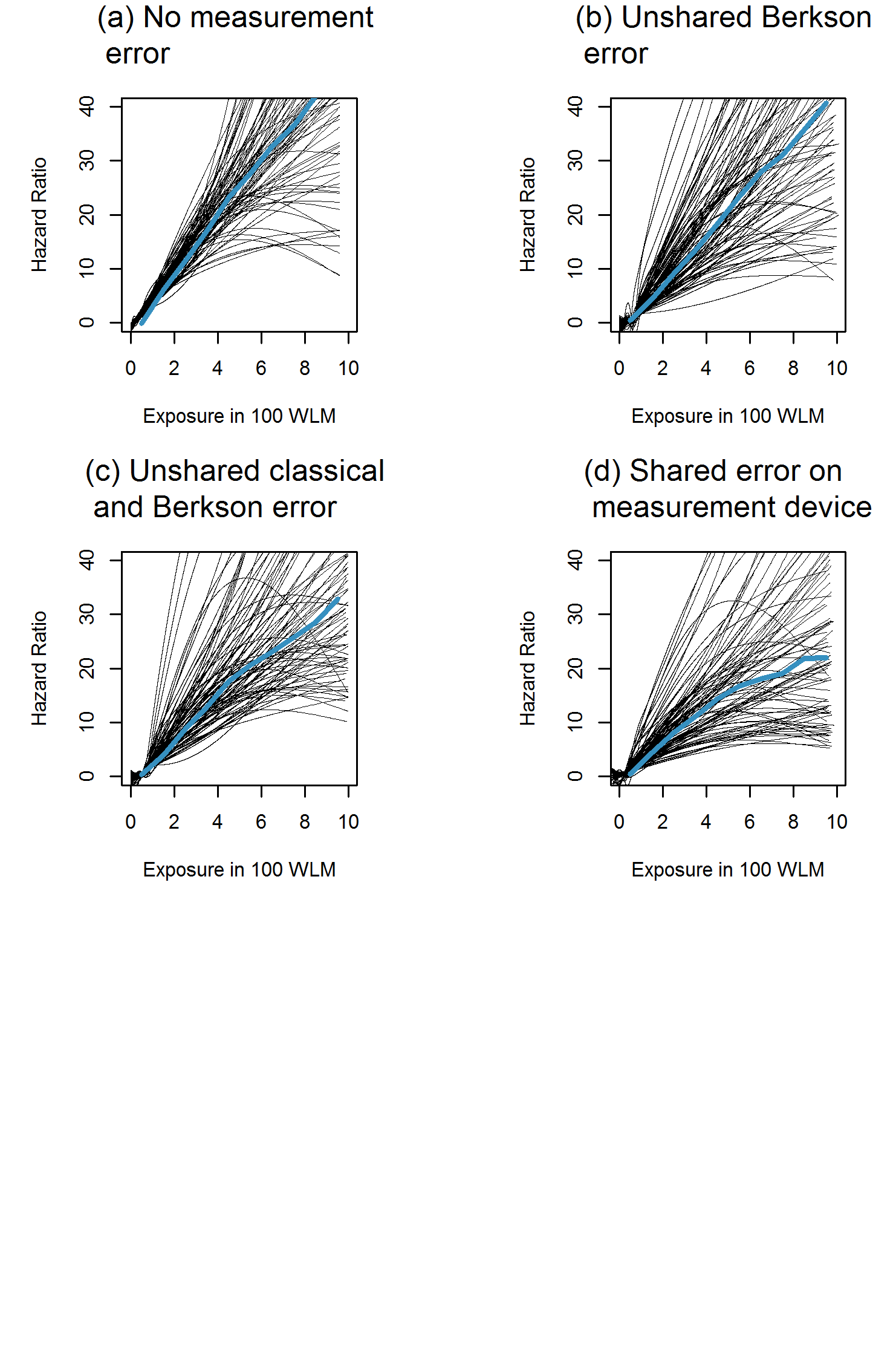

Supplement: S3 Fig — (a) M0, i.e., no measurement error (b) M1, i.e., unshared and homoscedastic Berkson error, (c) M9, i.e., unshared error of Berkson and classical type (d) M10, i.e., heteroscedastic error with a shared classical component describing the imprecision of the measurement device and (e) M11, i.e., heteroscedastic error with a shared Berkson component describing individual worker practices. (TIFF) [file pone.0190792.s003.tiff]
