# Supplementary material for: Shared and unshared exposure measurement error in occupational cohort studies and their effects on statistical inference in proportional hazards models
Source: PLoS One. 2018 Feb 6;13(2):e0190792. doi: 10.1371/journal.pone.0190792 (PMC5800563; doi:10.1371/journal.pone.0190792)
Supplement: S1 File — (PDF) [file pone.0190792.s004.pdf]

A more detailed presentation of measurement models $\mathcal{M}_3$, $\mathcal{M}_4$, $\mathcal{M}_5$, $\mathcal{M}_6$, $\mathcal{M}_7$ and $\mathcal{M}_8$

Measurement error shared between individuals

A Berkson model describing measurement error shared for all subjects that belong to group j can be written as

$$\mathcal{M}_3 : X_{ij}(t) = Z_j(t) \cdot U_j(t),$$

where $E(U_j(t)|Z_{ij}(t)) = 1$. The corresponding classical measurement error model is

$$\mathcal{M}_4 : Z_{ij}(t) = X_{ij}(t) \cdot U_j(t),$$

where $E(U_j(t)|X_{ij}(t)) = 1$.

Measurement error shared within individuals

For measurement error shared within individuals, one can derive the Berkson model

$$\mathcal{M}_5 : X_{ij}(t) = Z_j(t) \cdot U_i,$$

where $E(U_i|Z_j(t)) = 1$ and the classical measurement model

$$\mathcal{M}_6 : Z_{ij}(t) = X_{ij}(t) \cdot U_i,$$

where $E(U_i|X_{ij}(t)) = 1$. In contrast to models $\mathcal{M}_3$ and $\mathcal{M}_4$, the measurement error term U_i in these latter models does not depend on time t and is supposed to be the same for all years of exposure of subject i.

Measurement error shared both between and within individuals We assume a Berkson model of the form

$$\boldsymbol{\mathcal{M}_7:} X_{ij}(t) = Z_j(t) \cdot U_j,$$

where $E(U_j|Z_j(t)) = 1$ and a classical measurement model

$$\boldsymbol{\mathcal{M}_8:} Z_{ij}(t) = X_{ij}(t) \cdot U_j,$$

where $E(U_j|X_{ij}(t)) = 1$. In models $\boldsymbol{\mathcal{M}_7}$ and $\boldsymbol{\mathcal{M}_8}$, the measurement error term U_j does neither depend on time t nor on subject i, but only on the group j a subject belongs to. Consequently, the same error component is assigned for all times t a subject i belonging to group j received an exposure.
