# Supplementary material for: Shared and unshared exposure measurement error in occupational cohort studies and their effects on statistical inference in proportional hazards models
Source: PLoS One. 2018 Feb 6;13(2):e0190792. doi: 10.1371/journal.pone.0190792 (PMC5800563; doi:10.1371/journal.pone.0190792)
Supplement: S2 File — (PDF) [file pone.0190792.s005.pdf]

Complementary results

Results for alternative values of risk coefficients

Table 4 shows risk estimates and overall 95% credible intervals in the Cox proportional hazards model with a risk coefficent of $\beta = 5$.

Similarly to the results presented in Table 1 and Table 2, we observe more relative

Model	Type of sharing	Type of error	Error variance	$\hat{\beta}$	$CI_{95\%}$	Relative bias	Coverage rate
$\boldsymbol{\mathcal{M}_1}$	unshared	Berkson	0.1	4.15	[3.81; 4.47]	-0.17	0
			0.9	2.31	[1.79; 2.82]	-0.54	0
$\boldsymbol{\mathcal{M}_2}$		classical	0.1	3.85	[3.25; 4.25]	-0.23	0
			0.9	1.23	[0.51; 1.90]	-0.75	0
$\boldsymbol{\mathcal{M}_3}$	between	Berkson	0.1	4.11	[3.68; 4.47]	-0.18	0
			0.9	2.30	[1.74; 2.70]	-0.54	0
$\boldsymbol{\mathcal{M}_4}$		classical	0.1	3.87	[3.21; 4.29]	-0.23	0
			0.9	1.29	[0.51; 1.94]	-0.74	0
$\boldsymbol{\mathcal{M}_5}$	within	Berkson	0.1	3.04	[2.29; 3.44]	-0.39	0
			0.9	1.13	[0.86; 1.51]	-0.77	0
$\boldsymbol{\mathcal{M}_6}$		classical	0.1	2.86	[2.18; 3.33]	-0.43	0
			0.9	0.68	[0.33; 1.02]	-0.86	0
$\boldsymbol{\mathcal{M}_7}$	both	Berkson	0.1	3.19	[2.50; 3.78]	-0.36	0
			0.9	1.19	[0.70; 1.67]	-0.76	0
$\boldsymbol{\mathcal{M}_8}$		classical	0.1	3.10	[2.39; 3.64]	-0.38	0
			0.9	0.91	[0.38; 1.52]	-0.82	0
$\boldsymbol{\mathcal{M}_0}$	none	none	0	4.96	[4.67; 5.24]	-0.01	0.95

Table 1. Average posterior median ($\hat{\beta}$), overall 95% credible intervals ($CI_{95\%}$), relative bias and coverage rate for 100 data sets generated according to the Cox proportional hazards model $\boldsymbol{\mathcal{D}_2}$, a measurement model among $\boldsymbol{\mathcal{M}_0}$ to $\boldsymbol{\mathcal{M}_8}$ and a true risk coefficient of $\beta = 5$ per 100 WLM

bias in risk estimates and smaller coverage rates when exposure uncertainty is shared within workers than exposure uncertainty shared between workers or unshared uncertainty. Moreover, we observe relative bias in risk estimates for the Cox model that is generally larger when the true risk coefficient is $\beta = 5$ compared to $\beta = 2$ (see Table 1 in the main paper).

Likewise, Table 5 shows that the relative bias in risk estimates in the Excess Hazard Ratio (EHR) model is generally smaller when the true risk coefficient is $\beta = 2$ compared

to $\beta = 5$ (see Table 2 in the main paper). Regardless of the value of the true risk

Model	Type of sharing	Type of error	Error variance	$\hat{\beta}$	$CI_{95\%}$	Relative bias	Coverage rate
$\boldsymbol{\mathcal{M}_1}$	unshared	Berkson	0.1	1.99	[1.07; 3.26]	-0.01	0.98
			0.9	2.02	[1.09; 3.34]	0.01	0.97
$\boldsymbol{\mathcal{M}_2}$		classical	0.1	1.99	[1.04; 3.27]	- 0.01	0.96
			0.9	1.69	[0.85; 2.97]	- 0.16	0.86
$\boldsymbol{\mathcal{M}_3}$	between	Berkson	0.1	2.00	[1.07; 3.41]	0.00	0.94
			0.9	1.97	[1.03; 3.24]	- 0.02	0.97
$\boldsymbol{\mathcal{M}_4}$		classical	0.1	1.94	[1.01; 3.24]	- 0.03	0.94
			0.9	1.73	[0.89; 3.03]	- 0.14	0.84
$\boldsymbol{\mathcal{M}_5}$	within	Berkson	0.1	1.96	[1.07; 3.20]	- 0.02	0.97
			0.9	1.79	[0.88; 3.05]	- 0.11	0.87
$\boldsymbol{\mathcal{M}_6}$		classical	0.1	1.96	[1.02; 3.41]	- 0.02	0.93
			0.9	1.29	[0.60; 2.28]	- 0.36	0.41
$\boldsymbol{\mathcal{M}_7}$	both	Berkson	0.1	1.95	[1.02; 3.26]	- 0.03	0.95
			0.9	1.72	[0.86; 3.05]	- 0.14	0.88
$\boldsymbol{\mathcal{M}_8}$		classical	0.1	1.95	[1.00; 3.33]	- 0.03	0.92
			0.9	1.27	[0.56; 2.34]	- 0.37	0.43
$\boldsymbol{\mathcal{M}_0}$	none	none	0	2.00	[1.08; 3.35]	0.00	0.97

Table 2. Average posterior median ($\hat{\beta}$), overall 95% credible intervals ($CI_{95\%}$), relative bias and coverage rate for 100 data sets generated according to the EHR model $\boldsymbol{\mathcal{D}_1}$, a measurement model among $\boldsymbol{\mathcal{M}_0}$ to $\boldsymbol{\mathcal{M}_8}$ and a true risk coefficient of $\beta = 2$ per 100 WLM

coefficient and the type of measurement error, we observe the relative bias introduced by measurement error to be smaller in the EHR model than in the Cox model.

S1 and S2 Figs show the exposure-response curves estimated via cubic splines for the Cox model with $\beta = 5$ and the EHR model with $\beta = 2$, respectively. These figures and the results in Table 6 confirm that error structures with shared measurement error can introduce a strong attenuation of the exposure-response relationship in the Cox model, but not in the EHR model. In contrast to the results for the Cox model with $\beta = 2$ shown in Table 3, the risk coefficients for low exposures in the Cox model with $\beta = 5$ are never overestimated. It is also interesting to note that DIC values indicate for the three heteroscedastic measurement models ($\boldsymbol{\mathcal{M}_9}$, $\boldsymbol{\mathcal{M}_{10}}$ and $\boldsymbol{\mathcal{M}_{11}}$) that the EHR model is the better fitting disease model when failure times are generated according to the Cox model with a risk coefficient of $\beta = 5$, whereas this tendency was less pronounced for

**Disease model**	**Model $\mathcal{M}_0$** No error	**Model $\mathcal{M}_1$** Unshared Berkson error	**Model $\mathcal{M}_9$** Unshared heteroscedastic Berkson and classical error	**Model $\mathcal{M}_{10}$** Heteroscedastic shared device	**Model $\mathcal{M}_{11}$** Heteroscedastic worker practices
**Data generated according to the Cox model ($\mathcal{D}_2$) with $\beta = 5$**					
Risk estimate $\hat{\beta}$ in the linear Cox model ($\mathcal{D}_2$)	4.96 [4.68; 5.23]	3.62 [3.23; 4.02]	2.28 [1.78; 2.64]	1.00 [0.25; 1.85]	1.14 [0.82; 1.50]
Risk estimates in the piecewise-linear Cox model ($\mathcal{D}_6$)					
$\hat{\beta}_1$ (under 100 WLM)	4.94 [4.58; 5.32]	4.67 [4.29; 5.05]	4.21 [3.87; 4.56]	4.39 [3.86; 4.83]	3.76 [3.44; 4.07]
$\hat{\beta}_2$ (over 100 WLM)	4.99 [4.37; 5.56]	2.45 [1.77; 3.14]	0.96 [0.54; 1.42]	0.20 [-0.47; 0.69]	0.26 [-0.08; 0.59]
$\text{DIC}_{EHR} < \text{DIC}_{Cox}$	0%	0%	91 %	100%	100%
Difference in DIC	-757.38	-370.12	105.73	619.48	461.41
**Data generated according to the EHR model ($\mathcal{D}_1$) with $\beta = 2$**					
Risk estimate $\hat{\beta}$ in the in the linear EHR model ($\mathcal{D}_1$)	1.97 [1.08; 3.20]	2.03 [1.09; 3.44]	1.90 [1.01; 3.29]	1.61 [0.71; 3.18]	1.75 [0.90; 3.16]
Risk estimates in the piecewise-linear EHR model ($\mathcal{D}_5$)					
$\hat{\beta}_1$ (under 100 WLM)	1.93 [0.79; 3.66]	2.09 [0.81; 4.18]	1.95 [0.66; 3.94]	2.28 [1.00; 4.36]	1.92 [0.68; 3.95]
$\hat{\beta}_2$ (over 100 WLM)	2.12 [0.56; 4.24]	1.99 [0.47; 4.43]	1.91 [0.49; 4.07]	0.95 [0.01; 3.25]	1.57 [0.30; 3.66]
$\text{DIC}_{Cox} < \text{DIC}_{EHR}$	0%	1%	1%	0%	0%
Difference in DIC	20.93	22.00	21.36	37.39	20.56

Table 3. Comparison of risk estimates when data are generated according to different disease and measurement models. $\text{DIC}_{EHR} < \text{DIC}_{Cox}$ gives the percentage of realisations for which the Deviance Information Criterion (DIC) was smaller for the Excess Hazard Ratio (EHR) model when the true model was the Cox model and vice versa for $\text{DIC}_{Cox} < \text{DIC}_{EHR}$. The difference in DIC is calculated as difference between the EHR model and the Cox model.

model $\mathcal{M}_9$ when the true risk coefficient was $\beta = 2$ (see Table 3 in the main paper).

Comparison of results conducted by Bayesian and by frequentist inference

Table 4. Results on 100 data sets generated according to the Cox model $\mathcal{D}_2$, a measurement model among $\mathcal{M}_0$ to $\mathcal{M}_8$ and a true risk coefficient of $\beta = 2$ per 100 WLM: Comparison of the average Bayesian posterior median ($\hat{\beta}^{Bayes}$) and the average maximum likelihood estimate ($\hat{\beta}^{Likelihood}$) of β. The percentage of non-converged data sets indicates the relative frequency with which the Maximum Likelihood inference did not converge and $\hat{\beta}^{Bayes}$ on non-converged data sets gives information on the average Bayesian posterior median ($\hat{\beta}^{Bayes}$) estimated for the data sets for which Maximum Likelihood inference did not converge.

Model	Type of sharing	Type of error	Error variance	Bayesian inference $\hat{\beta}$	Bayesian inference Relative bias	Likelihood inference $\hat{\beta}$	Likelihood inference Relative bias
$\mathcal{M}_1$	unshared	Berkson	0.1	1.81	-0.10	1.83	-0.09
			0.9	1.25	-0.38	1.27	-0.37
$\mathcal{M}_2$		classical	0.1	1.75	-0.13	1.78	-0.11
			0.9	0.83	-0.59	0.84	-0.58
$\mathcal{M}_3$	between	Berkson	0.1	1.82	-0.09	1.85	-0.08
			0.9	1.25	-0.38	1.26	-0.37
$\mathcal{M}_4$		classical	0.1	1.75	-0.13	1.78	-0.11
			0.9	0.80	-0.60	0.82	-0.59
$\mathcal{M}_5$	within	Berkson	0.1	1.45	-0.28	1.47	-0.27
			0.9	0.76	-0.62	0.76	-0.62
$\mathcal{M}_6$		classical	0.1	1.33	-0.34	1.36	-0.32
			0.9	0.39	-0.81	0.39	-0.81
$\mathcal{M}_7$	both	Berkson	0.1	1.46	-0.27	1.47	-0.27
			0.9	0.77	-0.62	0.78	-0.61
$\mathcal{M}_8$		classical	0.1	1.42	-0.29	1.44	-0.28
			0.9	0.49	-0.76	0.50	-0.75
$\mathcal{M}_0$	none	none	0	1.96	-0.02	2.00	-0.00

Table 4 shows results on the impact of different types of measurement error on likelihood-based inference for the Cox model with a risk coefficient of $\beta = 2$, which we conducted in the R package *phreg*.

Attenuation of the exposure-response relationship for additive measurement error

Finally, we conducted inference for additive measurement error in the EHR model with a risk coefficient of $\beta = 5$ to be able to compare the effects of additive measurement error and multiplicative measurement error on the exposure-response relationship. The results, presented in S3 Fig, show that an additive error structure induces an attenuation of the exposure response relationship that is comparable to the attenuation introduced by a multiplicative error structure. Note that it was impossible to generate data for measurement model $\mathcal{M}_{11}$ in the case of additive error as it did not respect the necessary condition $h_i(t) = 1 + \beta X_i^{\text{cum}}(t) > 0$ and it was impossible to generate piecewise-exponential random variables with negative rates.
